# Lifestyle factors and psychological well-being: 10-year follow-up study in Lithuanian urban population

**DOI:** 10.1186/s12889-022-13413-4

**Published:** 2022-05-19

**Authors:** Laura Sapranaviciute-Zabazlajeva, Lolita Sileikiene, Dalia Luksiene, Abdonas Tamosiunas, Ricardas Radisauskas, Irena Milvidaite, Martin Bobak

**Affiliations:** 1 Health Psychology Department, Medical Academy, Lithuanian University of Health Sciences, Kaunas, Lithuania; 2 Institute of Cardiology, Medical Academy, Lithuanian University of Health Sciences, Sukileliu av. 15, LT-50162 Kaunas, Lithuania; 3 Institute of Epidemiology and Health care, University College London, London, UK

**Keywords:** Lifestyle behaviour, Psychological well-being, Follow-up

## Abstract

**Background:**

Several lifestyle behaviours, including physical activity, smoking, alcohol consumption, nutrition habits, and social activity have been associated with psychological well-being (PWB). However, their effect on PWB prospectively has been less studied. The aim of the present study was to evaluate the influence of lifestyle factors on higher future PWB during the 10-year follow-up of middle-aged and elderly urban population.

**Methods:**

In the baseline survey (2006 to 2008), 7115 men and women 45–72 years of age were examined within the framework of the international study Health, Alcohol and Psychosocial Factors in the Eastern Europe (HAPIEE). In the follow-up survey (in 2016), which was performed among all 6210 participants who survived till that year, 4266 individuals participated responding to postal questionnaires. PWB was assessed by a CASP-12 questionnaire. The lifestyle behaviours, including smoking and nutrition habits, alcohol consumption, social and physical activity, were evaluated by the questionnaire. Multivariable logistic regression models were applied for statistical data analysis.

**Results:**

After accounting for several potential confounders, healthy levels of lifestyle behaviours were associated with higher PWB after 10-year follow-up. Never-smokers in men and former smokers in women had higher PWB by 43 and 67% odds respectively in comparison with smokers. Physical activity in women and high social activity both in men in women was positively related to higher PWB. More frequent fresh vegetable and fruit consumption was associated with higher odds of higher PWB (odds ratio 1.57 in men and 1.36 in women, *p* < 0.05) compared to less frequent consumption of such food groups. Dose-response relationship between increasing number of healthy lifestyle factors and higher PWB was determined both in men and women.

**Conclusions:**

Lifestyle factors such as never smoking and former smoking, high social activity, and more frequent fresh vegetable and fruit consumption increased the odds of higher PWB over 10 years of follow-up in men and women groups. The increase of the protective health behaviour score was directly associated with the odds of higher PWB.

## Background

Lifestyle risk factors such as physical inactivity, smoking, unhealthy nutrition, harmful alcohol consumption have been associated with higher all-cause mortality and cardiovascular disease (CVD) morbidity [1–3]. In turn, promotion of healthy lifestyle behaviour, including non-smoking, limiting alcohol consumption, retaining optimal physical activity and body weight, adhering to healthy diet, is an effective approach for prevention of CVD and other chronic non-communicable diseases [4].

The incidence of mental disorders in the European region was estimated 517 cases per 100,000 population in 2013 [5]. The incidence of mental disorders among the countries in the region has been changing during the period of 10 years, some of them showing tendencies to go down, as it was in Estonia (from 2119 cases per 100,000 population in 2006, to 1966 cases per 100, 000 population in 2016) and Slovakia (from 1842 cases per 100,000 population in 2006, to 1365 cases per 100,000 population in 2016), while others showed the tendency to increase, e. g., in Germany (from 1416 cases per 100,000 population in 2006, to 1727 cases per 100,000 population in 2016), the Czech Republic (from 1089 cases per 100,000 population in 2006, to 1129 cases per 100,000 population in 2016), Latvia (from 466 cases per 100,000 population in 2006, to 478 cases per 100,000 population in 2016) [5]. The incidence of mental disorders in Lithuania during the period of 2006–2016 has been increasing from 261 to 319 cases per 100,000 population [6]. Prevalence of depression among Lithuanian inhabitants has been increasing in the period from 2014 (4.7%) to 2019 (7.0%); it was more prevalent among women (6.1 and 8.7%, respectively) compared to men (2.9 and 5.0%, respectively) [5]. Standardized mortality rates from mental and behavioural disorders per 100,000 population have been growing from 2.22 in 2006 to 2.9 in 2016 and increased up to 5.17 in 2019 [6].

Lifestyle behaviours are not only linked to physical health, but also to mental health including mental distress, depression and psychological well-being (PWB) [7–9]. PWB is considered to be a multidimensional construct, indicating not only the absence of mental distress, but also encompassing both hedonic and eudaimonic aspects of well-being [10, 11]. Results of recent studies have demonstrated that some lifestyle habits such healthy nutrition habits, were negatively associated with mental distress and positively with improved mental health and good cognitive function [8], but unhealthy diet was positively associated with mental distress [7]. In the analysis of the associations of lifestyle behaviours with mental health, the most common approach is to analyse the negative mental health outcomes such as anxiety, depressive symptoms, psychological distress [12, 13]. Compared to the results of epidemiological studies related to negative mental health outcomes, there is less evidence from the epidemiological studies of the effect of lifestyle factors on positive health outcomes, including positive PWB and happiness [14, 15].

Most of the previous studies examining the associations between lifestyle behaviours and mental health have only included one or two lifestyle factors: nutrition aspects, physical activity, alcohol consumption and other factors [9, 16, 17]. Since lifestyle factors frequently coexist, it is necessary to study the interrelationship between a greater quantity of lifestyle factors and mental health.

Results of most epidemiologic studies, including meta-analyses and systematic reviews which support a significant relationship between lifestyle factors and mental health, are often based on cross-sectional studies and much smaller numbers of longitudinal studies [7, 12, 15, 18, 19]. The nature of epidemiological studies investigating the links between health behaviour and mental health, including PWB, has been steadily changing from cross-sectional to longitudinal analysis [20]. Our previous analysis of the connection between healthy lifestyle and PWB in the sample of the Lithuanian population aged 45–72 also used a cross-sectional study design [21].

The aim of the present study is to evaluate the influence of lifestyle factors (smoking, alcohol consumption, physical activity, social activity, and nutrition status) on higher PWB during the 10-year follow-up in a large representative sample of Lithuanian middle-aged and elderly urban population.

## Methods

### Study sample

We used data from the Health, Alcohol and Psychosocial Factors in Eastern Europe (HAPIEE) study, a population-based urban cohort study conducted in Kaunas (Lithuania) [22]. Baseline data collection has been carried out between 2006 and 2008. A sample of Kaunas men and women (10,980 individuals) aged 45–72 years, stratified by gender and 5-year age groups, was randomly selected from the Kaunas population register. The response rate was 65%, which meant that 7100 respondents participated in the survey. A postal follow-up survey was conducted in 2016 among 6210 individuals who participated in the baseline survey and survived till April of 2016. During the follow-up survey, 1793 male and 2473 female were successfully re-examined with a response rate of 68.7%. The 3, 267 individuals (1, 472 men and 1795 women) who were re-examined were included in the analytical sample, which means that they had all the variables used in the logistic regression models for both the baseline and the follow-up surveys.

HAPIEE study received ethical approval by the Kaunas Regional Biomedical Research Ethics Committee (11 January 2005; No. 05/09) and by the Ethics Committee at the University College London. All participants signed the form of informed consent to participate in the survey and allowed to use their medical documents during follow-up.

### Sociodemographic factors

Sociodemographic factors were determined at the baseline survey using a standard questionnaire (age, marital status, education, employment status, number of children, and membership in social organizations) [22].

Social activity was determined by evaluating the respondents’ answers to the questions whether they often eat outside their homes (at a restaurant, cafe); attend sports events, go to theatres, cinemas, concerts, retirement clubs, clubs of special interests, participate in the activities of the church, maintain regular contact with relatives and friends [23]. Responses to the questions ranged from 0 to 5. Responses to 6 questions were summed up to create a score ranging from 0 to 30, where higher scores indicated higher social activity. The respondents were ranked from the lowest to the highest values and divided into three equal groups (tertiles) according to their social activity.

### Psychological measures

PWB both at baseline and follow-up surveys was employed for the present analysis. PWB was assessed by a Control Autonomy Self-realization and Pleasure (CASP-12) questionnaire [24]. For this measurement, participants were presented with a list of 12 statements which described their lives or how they feel. Their answers on a 4-point scale were ranged from “never” to “often”, resulting in scores ranging from 12 to 48. The internal consistency of CASP-12 scale was good (Cronbach’s alpha = 0.74). PWB scores of participants were classified into the category of a higher PWB group if the scores were equal to median or higher (baseline survey: > 40 in men and > 38 in women; follow-up survey: > 36 in men and > 35 in women). Participants with PWB scores lower than median were classified into the group of a lower PWB. The change of PWB over 10 years was measured by evaluating the changes in CASP-12 score categories between baseline and follow-up surveys: 1. No change (higher or lower PWB at both surveys); 2. Improved PWB (lower PWB at baseline survey and higher PWB at follow-up survey); 3. Deteriorated PWB (higher PWB at baseline survey, and lower PWB at follow-up survey).

We measured depressive symptoms using the Center for Epidemiological Studies Depression Scale-10 (CESD-10) [25]. The CESD-10 is a 2-point scale (1 (yes) and 0 (no)) assessing the extent to which individuals experienced 10 depressive symptoms during the prior week. The possible total score varies between 0 and 10. Participants scoring > 4 were considered to have depressive symptoms [26]. The CESD-10 scale showed rather good internal consistency (Cronbach’s alpha = 0.72).

### Lifestyle factors

Lifestyle factors were evaluated using a standard questionnaire and some anthropometric measurements.

Smoking status was classified as never smoking, former smoking and current smoking. Current smokers were individuals who regularly smoked at least 1 cigarette per day.

Alcohol consumption frequency was categorised as never, less than 1 time per month, 1 to 3 times per month, once per week, 2 to 4 times per week, every day. Respondents reported the quantity of spirits, beer, and wine usually consumed per week. According to the recommendations of the Handbook of Alcoholism [27], the responses were converted into units of alcohol, assuming the measure of the spirits consumed to be standard alcohol units (drinks) (SAU): a bottle (0.5 L) of beer to be two SAU and a bottle of wine (0.7 L) to be six SAU.

In order to assess the physical activity of the participants in their leisure time, 5 questions were asked. Physical activity was determined by the mean length of time spent per week during leisure time in autumn-winter and spring-summer seasons, such as gardening, maintenance of the house, and other physical activities. The respondents were ranked from the lowest to the highest values and divided into three equal groups (tertiles) according to their physical activity during leisure activities. The first tertile cut-off (max) is 10 hours. For this reason, we used this cut-off to identify insufficient physical activity.

Intake of 20 food groups was assessed using our study food frequency questionnaire. The food groups were boiled vegetables, candies, cakes and chocolate, cheese, curd cheese, chicken, eggs, fish, fresh carrots, other fresh vegetables, fresh fruit, meat, natural juice, potatoes, porridges and cereals, sausage. The questionnaire included questions about the frequency of consumption of fresh carrots, other fresh vegetables, natural juices, and fresh fruit in different seasons of the year (in summer and autumn and in winter and spring). Mean values of the use of fresh carrots, other fresh vegetables, fresh fruit and natural juice in summer and autumn and in winter and spring were calculated, and 16 food groups were included in the final analysis. In the food frequency questionnaire, there were six possible categories of answers for each food group: less than 2–3 times per month or never; 2–3 times per month; once per week; 2–3 times per week; 4–6 times per week; every day. Factor analysis was used to reduce the number of food groups. Data on explanatory factor analysis were presented in our previous publications [21, 28]. The factor analysis revealed new five-factor food groups: consumption of sweets, consumption of porridge and cereals, consumption of chicken and fish, and consumption of potatoes, meat, boiled vegetables, and eggs. Each new food group was constructed as dichotomous-dependent variable by dividing factor scores into groups: 1. more frequent than average consumption of food items in the group; 0 – less frequent than average consumption of food items.

### Objective measurements

Objective measurements (blood pressure, height, and body weight), and biochemical analyses (high-density lipoprotein (HDL) cholesterol, low-density lipoprotein (LDL) cholesterol, fasting glucose, and triglyceride) were determined at baseline survey.

Weight and height were measured with a calibrated medical scale. Body mass index (BMI) was calculated as the weight in kilograms divided by the height in meters squared (kg/m^2^). We divided study participants into groups: group with normal weight (BMI 18.5–24.99 kg/m^2^), overweight (BMI 25.0–29.99 kg/m^2^), and obesity (BMI ⩾30.0 kg/m^2^). Insufficient weight was defined as BMI < 18.5 kg/m^2^ [29].

Coronary heart disease (CHD) was determined using the following criteria: (1) a documented history of myocardial infarction (MI) and/or ischemic changes on electrocardiogram (ECG) coded by Minnesota codes (MC) 1–1 or 1–2 [30]; (2) angina pectoris was defined by G. Rose’s questionnaire (without history of MI and/or MC 1–1 or 1–2) [31]; (3) ischemic changes on ECG coded by MC 1–3, 4–1, 4–2, 4–3, 5–1, 5–2, 5–3, 6–1, 6–2, 7–1, or 8–3 (without MI and/or MC 1–1, 1–2 and without angina pectoris).

### Protective health behaviour score

Using the experience and examples from other studies, we also evaluated the combined impact of healthy lifestyles on PWB by constructing a protective health behaviour score (PHBS) [32, 33]. In our study, we used 6 lifestyle factors for the calculation of PHBS. Each factor was dichotomized into two levels: a healthy level of lifestyle behaviour and a less healthy or unhealthy lifestyle behaviour. The threshold of dividing lifestyle factors into levels was based on the data from other studies and our own experience [33–35]. Study participants scored one point for each of the following healthy level of lifestyle patterns: non-smoking (never and former smokers), having normal BMI, being physically active, being a moderate alcohol drinker (drinking alcohol once per week or less frequently), being moderately or highly socially active, eating fruit and vegetables more frequently. Conversely, participants scored 0 point for each unhealthy level of lifestyle behaviour. The total possible PHBS varied between 0 and 6 points.

### Statistical analysis

All data were analysed using IBM SPSS (Version 20.0) software package. All analyses were performed separately for men and women. The distributions of lifestyle factors and PHBS groups were compared in PWB groups at baseline survey using chi-square and z tests. We also compared the proportions of lifestyle factors in groups of PWB change over 10 years (between group of improved PWB and group of deteriorated PWB) using the same statistical tests. Mean differences were tested using t-test. *P* < 0.05 values were considered statistically significant.

For multivariable analysis, we used all variables that were significantly associated with higher PWB over 10 years of follow-up in the univariate logistic regression analysis. Odds ratios (OR) and 95% confidence intervals (CI) were calculated for categories of all 6 lifestyle factors. Two models were assessed. Model 1 was adjusted for age (continuous), arterial hypertension (categorical), BMI, glucose level, triglycerides, HDL cholesterol, and LDL cholesterol (all continuous variables). Model 2 was adjusted for all the variables in Model 1 plus marital status, education, employment status, number of children, membership in social organizations, depressive symptoms, and CHD (all categorical). To assess the combined effect of lifestyle on higher PWB over 10 years of follow-up, similar two logistic regression models were assessed and OR and 95% CI calculated for PHBS groups. The reference category used was 0 to 2 points group of PHBS.

## Results

Table 1 shows the baseline characteristics of lifestyle factors according to the initial level of PWB differentiated by gender. Men and women with higher PWB were more socially and physically active. Also, they had healthier nutrition habits and were more likely to have a higher proportion of PHBS compared to responders with lower PWB.

**Table 1:** Baseline characteristics of lifestyle factors according to the initial level of PWB in gender groups

Lifestyle factors	Men		Women	
	Lower PWB	Higher PWB	Lower PWB	Higher PWB
**Smoking status,** %				
Current	27.5	22.3	10.2	8.3
Former	29.9	31.1	7.2	6.7
Never	42.6	46.5	82.6	84.9
**Cigarettes, number per day**, **mean (SD)**	16.9 (10.2)	16.5 (10.1)	8.5 (6.3)	8.6 (7.2)
**Alcohol consumption**, %				
Every day	6.3	4.3	0.9	1.1
2–4 times per week	14.6	18.2	1.5	3.0*
Once a week	17.6	16.5	7.1	8.0
1–3 times per month	34.0	35.4	30.1	36.6*
Less than once a month	22.2	22.0	52.4	46.9*
Never	5.3	3.6	7.9	4.4*
**Alcohol consumption, SAU per week, mean (SD)**	6.08 (10.9)	276.2 (5.31)	1.27 (2.02)	1.55 (2.18)**
**Physically active**, %	69.4	72.9	77.0	84.9***
**Physical activity in leisure time, hours per week, mean (SD)**	17.4 (12.0)	18.9 (12.6)*	19.1 (11.5)	21.4 (12.0)***
**Nutrition habits** %				
More frequent fresh vegetables, fruit consumption	48.8	57.9***	49.8	67.7***
More frequent sweets consumption	48.8	53.0	48.7	47.1
More frequent porridge, cereals, curd cheese consumption	34.6	40.2*	63.1	63.6
More frequent potatoes, meat, boiled vegetables, and eggs consumption	65.1	59.5*	45.6	41.5
More frequent chicken and fish consumption	52.5	57.3	47.1	52.5*
**Social activity, %**				
Low	34.3	23.9***	34.5	17.9***
Medium	37.2	32.1*	41.1	39.6
High	28.5	44.0***	24.3	42.5***
**Protective health behaviour score (PHBS), %**				
0	0.6	0.0	0.0	0.0
1	2.6	2.2	0.5	0.3
2	13.3	9.0**	5.3	1.8***
0–2	16.5	11.2**	5.8	2.1***
3	28.5	24.5	26.1	18.5***
4	34.3	37.1	35.9	33.5
5	17.6	23.4**	27.8	42.7***
6	3.1	3.8	4.4	8.9***

*SAU* standard alcohol units (drinks), *PWB* psychological well-being

*p < 0.05

**p < 0.01

***p < 0.001 compared to lower PWB group

PWB improved for 23% of men and for 26% of women and deteriorated for 71.6% of men and for 67.4% of women after 10 years.

Table 2 shows the distribution of lifestyle factors in the initial study according to the changes in PWB after 10 years. Smoking status and some dietary habits were found to differ significantly with respect to changes in PWB: after 10 years, there were more smokers and fewer never smokers among men whose PWB had worsened, as compared to men whose PWB had improved. Men and women with deteriorated PWB consumed chicken and fish more frequently compared to responders with improved PWB.

**Table 2:** Lifestyle factors in initial study and distribution of PWB after 10 years in gender groups

Lifestyle factors	Men		Women	
	Improved PWB	Deteriorated PWB	Improved PWB	Deteriorated PWB
**Smoking status,** %				
Current	21.7	26.6*	10.5	9.4
Former	27.9	29.9	8.6	6.8
Never	50.5	43.5*	80.9	83.8
**Cigarettes, number per day**, **mean (SD)**	17.7 (10.9)	16.3 (9.9)	7.5 (6.3)	8.7 (7.2)
**Alcohol consumption**, %				
Every day	5.7	5.3	1.0	1.2
2–4 times per week	14.5	17.8	2.9	2.6
Once a week	19.5	16.0	7.8	8.6
1–3 times per month	34.0	36.6	35.6	35.7
Less than once a month	21.4	20.5	47.0	46.6
Never	4.8	3.8	5.7	5.4
**Alcohol consumption, SAU per week, mean (SD)**	5.31 (7.21)	6.12 (10.9)	1.65 (2.28)	1.38 (2.36*)
**Physically active**, %	71.7	71.2	79.1	82.8
**Physical activity in leisure time, hours per week, mean (SD)**	18.6 (13.0)	17.7 (11.7)	19.9 (11.9)	20.8 (11.9)
**Nutrition habits** %				
More frequent fresh vegetables, fruit consumption	45.5	45.5	61.1	61.5
More frequent sweets consumption	50.2	51.1	50.7	48.7
More frequent porridge, cereals, curd, and cheese consumption	39.5	36.9	65.1	61.6
More frequent potatoes, meat, boiled vegetables, and eggs consumption	63.8	61.5	43.4	44.3
More frequent chicken and fish consumption	51.0	57.3*	46.1	51.7*
**Social activity, %**				
Low	25.2	27.8	23.8	21.9
Moderate	37.6	32.5	37.3	41.6
High	37.1	39.7	38.9	36.5

*SAU* standard alcohol units (drinks), *PWB* psychological well-being

*p < 0.05

**p < 0.01

***p < 0.001 compared to improved PWB group

Table 3 shows the OR of higher PWB over 10 years of follow-up according to the lifestyle factors. After adjustment for age and biological factors (Model 1), some lifestyle factors such as never smoking in men and former smoking in women significantly increased the odds of higher PWB compared to current smokers. Moderate and high social activity, both in men and women, increased the odds of higher PWB compared to responders with low social activity level. More frequent fresh vegetable and fruit consumption increased the odds of higher PWB for men and women. However, some nutrition habits, such as more frequent consumption of sweets increased the odds of higher PWB in men. More frequent consumption of potatoes, meat, boiled vegetables, and eggs decreased the odds of higher PWB in women. Physical activity increased the odds of PWB in women group. After additional adjustment for socioeconomic, social, and sociodemographic factors, depressive symptoms, and CHD (Model 2) lifestyle factors such as smoking habits (never smoking in men; former smoking in women), high social activity, and more frequent fresh vegetable and fruit consumption increased the odds of higher PWB over 10 years of follow-up in the groups of men and women. More frequent consumption of potatoes, meat, boiled vegetables, and eggs decreased the odds of higher PWB in women.

**Table 3:** OR of higher PWB over 10 years of follow-up according to lifestyle factors^a^

Lifestyle factors	MEN				WOMEN			
	Model 1		Model 2		Model 1		Model 2	
	*n* = 1472				*n* = 1795			
	OR	95% CI	OR	95% CI	OR	95% CI	OR	95% CI
Smoking status								
Current	1		1		1		1	
Former	1.25	0.93–1.69	1.30	0.95–1.78	**1.67**	**1.03–2.68**	**1.91**	**1.14–3.21**
Never	**1.54**	**1.17–2.05**	**1.43**	**1.07–1.92**	1.29	0.92–1.81	1.14	0.79–1.66
Alcohol consumption								
Every day	1		1		1		1	
2–4 times per week	1.27	0.75–2.14	1.20	0.69–2.06	0.93	0.31–2.81	0.77	0.23–2.57
Once per week	1.70	1.00–2.87	1.53	0.88–2.64	0.93	0.35–2.52	0.77	0.26–2.29
1–3 times per month	1.60	0.98–2.62	1.45	0.87–2.42	0.85	0.33–2.19	0.72	0.25–2.04
Less than once per month	1.43	0.85–2.41	1.44	0.84–2.48	0.76	0.30–1.96	0.72	0.25–2.03
Never	1.34	0.66–2.72	1.51	0.72–3.18	0.60	0.22–1.68	0.64	0.21–1.96
Physically active	1.00	0.79–1.27	1.03	0.81–1.32	**1.34**	**1.04–1.72**	1.23	0.94–1.63
Social activity								
Low	1		1		1		1	
Moderate	**1.42**	**1.08–1.87**	1.20	0.90–1.61	**1.38**	**1.07–1.79**	1.05	0.80–1.39
High	**2.20**	**1.67–2.91**	**1.48**	**1.08–2.02**	**2.50**	**1.91–3.28**	**1.68**	**1.24–2.29**
Nutrition status								
More frequent fresh vegetables and fruit consumption	**1.62**	**1.30–2.01**	**1.57**	**1.24–1.97**	**1.58**	**1.29–1.93**	**1.36**	**1.09–1.69**
More frequent sweets consumption	**1.25**	**1.01–1.55**	1.24	0.98–1.55	0.96	0.78–1.17	0.97	0.78–1.19
More frequent porridge, cereals, cheese consumption	1.08	0.86–1.35	1.10	0.87–1.39	0.98	0.80–1.20	1.02	0.82–1.27
More frequent potatoes, meat, boiled vegetables, eggs consumption	0.84	0.67–1.04	0.84	0.67–1.06	**0.80**	**0.65–0.97**	**0.80**	**0.65–0.99**
Chicken, fish consumption	1.01	0.82–1.25	1.03	0.83–1.29	0.91	0.75–1.10	0.87	0.71–1.08

Model 1 adjusted for age, biological factors (arterial hypertension, body mass index, glucose level, triglycerides, HDL cholesterol and LDL cholesterol). Model 2 adjusted for all the variables in Model 1 plus socioeconomic, social, and socio-demographic factors (marital status, education, employment status, number of children, and membership of social organization), depressive symptoms and CHD

Bold typeface indicates significance

*CHD* coronary heart disease, *CI* confidence interval, *HDL* high density lipoprotein, *LDL* low density lipoprotein, *OR* odds ratio, *PWB* psychological well-being

^a^lifestyle factors in the initial survey

Table 4 shows the OR of higher PWB over 10 years of follow-up according to the protective health behaviour score (PHBS) in the initial survey. The odds of higher PWB significantly increased with the increasing number of PHBS (Model 1). The odds of higher PWB were more than two times higher in men and women with four to six PHBS compared with those responders who scored 0 to 2 for PHBS. After additional adjustment (Model 2), the results were similar in the group of men, but in the group of women, the significance was determined only in the women with six PHBS compared to the women with zero to two PHBS.

**Table 4:** OR of higher PWB over 10 years of follow-up according to protective health behaviour score^a^

Protective health behaviour score (PHBS)	MEN				WOMEN			
	Model 1		Model 2		Model 1		Model 2	
	*n* = 1472				*n* = 1795			
	OR	95% CI	OR	95% CI	OR	95% CI	OR	95% CI
0–2	1		1		1		1	
3	**1.53**	**1.08–2.18**	1.27	0.87–1.84	1.52	0.85–2.72	1.20	0.63–2.26
4	**2.47**	**1.76–3.47**	**1.98**	**1.38–2.85**	**2.22**	**1.27–3.89**	1.41	0.76–2.61
5	**3.36**	**2.31–4.89**	**2.55**	**1.72–3.79**	**3.36**	**1.92–5.89**	1.85	1.00–3.42
6	**3.09**	**1.60–6.00**	**2.09**	**1.03–4.22**	**4.44**	**2.30–8.56**	**2.19**	**1.07–4.49**

Model 1 adjusted for age, biological factors (arterial hypertension, glucose level, triglycerides, HDL cholesterol and LDL cholesterol). Model 2 adjusted for all the variables in Model 1 plus socioeconomic, social, and socio-demographic factors (marital status, education, employment status, number of children, and membership of social organization), depressive symptoms and CHD

Bold typeface indicates significance

*CHD* coronary heart disease, *CI* confidence interval, *HDL* high density lipoprotein, *LDL* low density lipoprotein, *OR* odds ratio, *PWB* psychological well-being

^a^protective health behaviour score in the initial survey

## Discussion

This study explored the degree to which lifestyle behaviours, such as alcohol consumption, smoking, physical and social activity, and nutrition habits are associated with higher future PWH in a large representative sample of middle-aged and elderly Lithuanian urban population. Never smoking in men and former smoking in women, higher physical activity in women, high social activity and more frequent intake of vegetables and fruit in men and women were positively associated with higher PWB after adjusting for several covariates. Furthermore, a positive effect was observed between the baseline protective health behaviour score and higher PWB during 10-year follow-up.

Our study results extend those of previous research which also found positive relationship between lifestyle factors and good mental health, including higher PWB [12, 14, 36]. Importantly, our study shows that those associations were present even after statistical adjustment for several covariates such as biological factors, socioeconomic and sociodemographic factors, other mental health indicators (depressive symptoms) and chronic disease (coronary heart disease).

According to the WHO, healthy lifestyle means regular physical activity, no smoking, refusal of alcohol, healthy eating and avoiding being overweight. Such behavior should lead not only to better physical and mental health but also to well-being [37]. Recent prospective studies have found a two-way association between lifestyle and mental and emotional health and well-being [38]. Healthy lifestyle features can have a positive effect on the development of depression and anxiety, life satisfaction and self-perceived general mental health [39].

Changes in body weight could be one of the biological factors associated with well-being, mental health and higher PWB. According to Stranges et al., underweight is inversely associated with well-being and overweight is positively associated with well-being [36]. This may be due to the fact that persons who are underweight may have other metabolic or digestive disorders that can lead to a lack of certain essential substances and hormones in the body, which in turn can lead to certain mental health problems. These data suggest a U-shaped association between BMI and well-being, and, at the same time, overweight has a protective effect on emotional well-being. There are limited data on the association between obesity, mental health, and dietary habits. According to Meegan A.P., the association between nutritional quality and well-being has been identified and remains significant only in non-obese individuals. No association or symptoms of anxiety were observed among obese individuals [12]. In addition, Jacka et al. data suggest that an inverse causal association may explain the links between diet and mental health [40]. Thus, it is clear that the associations between nutrition, overweight and mental health are very complex. Further cohort prospective studies that would examine these associations and focus on the reverse causality hypothesis are needed to further determine the direction and underlying causal mechanisms of these associations.

The relationship between nutrition habits and mental health, including PWB, is quite extensively studied. Most of previous studies, similarly to our study, considered vegetables and fruit as a combined food group [9, 41]. Only some studies have assessed the impact of vegetables and fruit as an independent food group in the regression models [42, 43]. The findings from these studies demonstrate that the effect of consumption of vegetables on PWB was greater when compared to the consumption of fruit. The results of the exploratory factor analysis of the CoLaus / PsyCoLaus study (Lausanne, Switzerland) showed that the “Sweet-Dairy” diet (mainly chocolate, biscuits, pastries, cakes, butter, high-fat and low-fat dairy products, jam, and honey) was positively related to melancholic depression [44]. In contrast, our data show that the consumption of sweets was significantly related to higher PWB in men but not in women. We found that an increase in the frequency of consumption of potatoes, meat, boiled vegetables, and eggs was related to significantly lower odds of higher PWB in women. Our data contrasts with previous research showing that not only fresh vegetables and fruit but also processed vegetables and fruit (dried or canned) are associated with better mental health [45, 46]. Healthy eating patterns, such as those in the Mediterranean and some Nordic countries, which are characterized by high consumption of vegetables and fruits, whole grains, lean fish, and meat, have been associated with a reduced risk of depressive complaints [47]. Meanwhile, an unhealthy diet, characterized by increased consumption of processed meat, fatty foods, refined grains, high-sugar products, and alcohol, has been associated with an increased risk of depression and poorer mental health [48]. According to the data of other recent studies, among women and men high consumption of fruit and vegetables, moderate consumption of dairy, and moderate-high intake of meat were negatively associated with mental distress and improved mental health and cognitive function [8], whereas high fast-food and caffeine consumption was positively associated with mental distress [7].

Our data indicate that in men, compared to current smokers, being a never-smoker was associated with an increased probability of higher PWB in partially and fully adjusted models. Similar result was found among women when former smokers were compared to current smokers (OR = 1.67, *p* < 0.05 and OR = 1.91, *p* < 0.05 for partially and fully adjusted models respectively). In contrast, among 13,983 adults 16+ of age from England, reduced ORs of good mental well-being were determined among former smokers. The authors attributed this finding to the fact that smoking cessation may have been a consequence of the underlying disease rather than a preventive measure. Such an effect has not been taken into account by other researchers [36]. To date, most of the studies have shown that individuals who never smoked or stopped smoking demonstrate a significant increase in positive PWB compared to the individuals who continue smoking [49–51]. Other researchers have also found that smoking is linked to greater mental health problems. In a prospective study, subjects who continued to smoke had increased symptoms of depression, anxiety, and stress [51]. After quitting smoking, more time is made available for other lifestyle-related activities, such as physical or social activity, which are linked to better emotional well-being and better mental health. Further promotion of quitting smoking programs can help to stop smoking and thus improve not only a person’s physical but also his/her mental health [52].

Results from epidemiological studies of the relationship between alcohol consumption and mental health including PWB are controversial. Findings in some studies show a U-shaped association indicating a better PWB or lower risk of mental health outcomes among moderate drinkers of alcohol in comparison with abstainers or never drinkers and heavy drinkers [36, 53, 54]. In the study of New Zealand adults, drinkers of alcohol once a month or less, compared to drinkers up to 4 times per month, had significantly lower odds of optimal well-being in crude, partially adjusted and fully adjusted binary logistic regression models [49]. In contrast, other studies have not found significant association between alcohol consumption and well-being or found it only after applying crude but not adjusted regression models [15, 50]. Our study also revealed no significant relationship between alcohol consumption and PWB. However, the following tendency was observed: decreased frequency of alcohol consumption was associated with lower odds of higher PWB in comparison with everyday alcohol drinkers. This may be related to the fact that after 10 years, the subjects’ reduced alcohol consumption is associated with lifestyle changes due to other chronic health conditions that could lead to changes in both physical and mental health, and the same time, to a lower PWB.

Social activities, including engagement in cultural events, arts, sports, and other clubs, in relation to mental health and well-being are quite widely explored in epidemiological studies. Findings from 5338 adults examined in the UK showed that more arts engagement was associated with higher levels of well-being and social connectedness, lower odds of intense social loneliness and, in contrast, positively related with depression and intense emotional loneliness [55]. Mental activities, including making music, going to the cinema or theatres, reading books, were positively related with positive mental health at baseline survey but not at 1-year follow-up survey using matched data of German and Chinese students [14]. In the present study, moderate and high social activity (participation in clubs, visiting the church, theatres, restaurants, and other cultural events) was significantly related to higher future PWB both for men and women compared to low social activity individuals. One explanation could be that individuals who are socially active have greater physical activity, which has a positive effect on both physical and mental health and increases well-being. It cannot be ruled out that the traits of an individual study depend on genotype and character traits, and certain sociodemographic components such as marital status, education, and socioeconomic conditions. Frequent loneliness, lower education, lack of income, unemployment were associated with lower social activity and increased the risk of depression, at the same time worsening mental health and well-being [56–58].

The positive effect of physical activity on mental health is attributed to the release of endorphins which help to boost well-being and increase energy [59]. A higher level of physical activity has been linked with reduced risk of mental health outcomes and better PWB [14, 49, 60]. In young women (aged 18 to 29 years) and mature women (aged 30 years and older), high frequency of physical exercise was significantly associated with mental well-being. In young men but not in mature men, frequent exercise was negatively related with mental distress [7]. Our data indicates that among women physically active individuals showed significantly higher odds (OR = 1.34, *p* < 0.05) of better PWB in comparison with those people who were physically inactive but only in partially adjusted regression model. In men, no association between physical activity and future higher PWB was observed. This can be explained by the fact that most men worked physically and that higher physical activity could not have had a greater effect on higher PWB during the study period despite the older age of the men studied.

The combined effect of several risk factors on all-cause and CVD mortality has been quite widely explored and is associated with higher mortality rates [61, 62]. The relationship between healthy levels of biological factors and healthy lifestyle behaviours and both physical health and mental health was less intensively examined [34, 63, 64]. In the sample of 25,837 participants from the NutriNet-Sante (France) study, after a mean follow-up of 5 years, incidence of depressive symptoms in individuals having 5 healthy lifestyle indicators was significantly lower by 25% compared to individuals having 0–2 indicators [65]. Other recent studies have found that a higher healthy lifestyle score was associated with lower overall mortality [2, 3]. Our findings show that in men, a greater number of healthy lifestyle behaviour (3 to 6) was associated with significantly higher odds of higher PWB after 10-year follow-up in comparison with men having 0–2 healthy lifestyle behaviour factors with adjustment to several confounders in the regression model. In women, significant relationship between the score of healthy lifestyle behaviour and higher PWB was determined only when individuals having 0–2 and 6 such factors were compared.

### Strengths and limitations

Strengths of the present study are its prospective design, large sample size and wide age interval of study participants including middle-aged and elderly individuals (45–72 years at baseline). Other strengths are data collection using standardized and validated study methods, long follow-up period (from 2006 to 2008 to 2016) and many potential confounders included into statistical analyses (up to 13 variables in fully adjusted multivariate regression models). This study also has some limitations. First, we do not know exactly what diseases or additional health disorders our subjects had during the follow-up period, and what new risk factors for chronic diseases and harmful lifestyles emerged during that period, and for how long. Secondly, lifestyle behaviours and PWB were self-reported by study participants who may have been affected by recall bias and this could have resulted in overestimation or underestimation of the determined outcomes. Thirdly, we included traditional lifestyle behaviour factors in this study. Factors, especially the so-called non-traditional lifestyle factors (sleep duration, quality of sleep, sedentary lifestyle, and other factors), that were not included in our study could also be related to PWB. Finally, caution should be exercised when generalizing our findings to the Lithuanian population, as only Kaunas city residents were included in the random sample of our study.

## Conclusions

Our findings revealed that lifestyle factors, such as smoking habits (never smoking in men, former smoking in women), high social activity, and more frequent fresh vegetable and fruit consumption increased the odds of a higher PWB over 10 years of follow-up in men and women groups. More frequent consumption of potatoes, meat, boiled vegetables, and eggs decreased the odds of a higher PWB in women. Our study results suggest that the increase of the protective health behaviour score was directly associated with the odds of a higher PWB.

These results also suggest that maintaining and improving the mental health and PWB of the middle-aged and older population require the use of healthy lifestyle behavioural interventions and that promotion of healthy lifestyle should be a public health priority.

## Data Availability

All data generated or analyzed during this study are included in this article. The datasets used and/ or analysed during the current study are available from the corresponding author on reasonable request.

## Abbreviations

BMI: Body mass index
CHD: Coronary heart disease
CI: Confidence interval
CVD: Cardiovascular disease
ECG: Electrocardiogram
HDL: High-density lipoprotein
LDL: Low-density lipoprotein
MC: Minnesota codes
MI: Myocardial infarction
OR: Odds ratio
PHBS: Protective health behaviour score
PWB: Psychological well-being
SAU: Standard alcohol units
SD: Standard deviation
